# Supplementary material for: Solution structure of Titan-relevant aqueous ammonia by neutron diffraction
Source: Commun Chem. 2025 Aug 2;8:227. doi: 10.1038/s42004-025-01599-8 (PMC12318028; doi:10.1038/s42004-025-01599-8)
Supplement: Supplementary file 2 — Supplementary material [file 42004_2025_1599_MOESM2_ESM.pdf]

Solution Structure of Titan-Relevant Aqueous Ammonia by Neutron Diffraction

Mazin. Nasralla[1], Harrison Laurent[1], Oliver L.G. Alderman[2] and Lorna Dougan[1*]
[1]School of Physics and Astronomy, University of Leeds, Leeds, LS2 9JT, United Kingdom.
[2]ISIS Neutron and Muon source, Harwell Campus, Didcot, OX11 0QX, United Kingdom.
(*L.Dougan@leeds.ac.uk)
Dated: June 30, 2025)

Supplementary Information

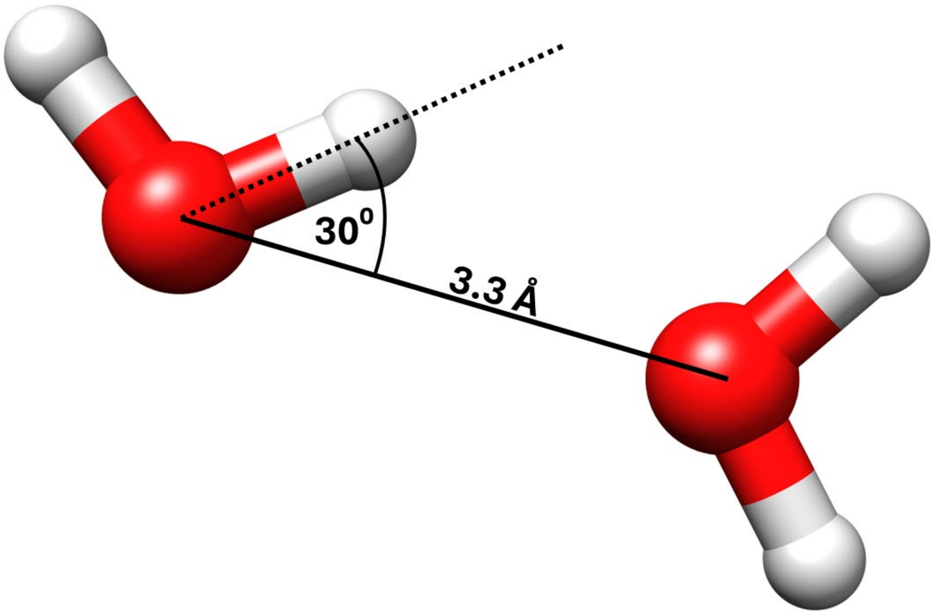

Fig. S1. Hydrogen bond boundary conditions

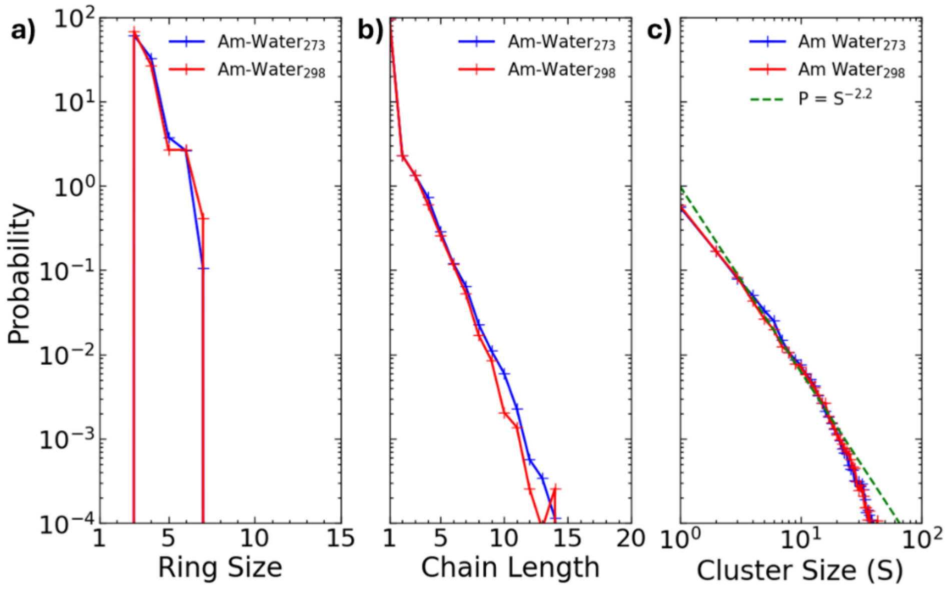

Fig. S2. The topology of the Ammonia network. These results show that the ammonia network is at best limited. In panel a) we report the size distribution of rings of hydrogen bonded ammonia molecules by the shortest path. In b) we illustrate the size distribution of linear chains of hydrogen bonded water molecules that fulfil a shortest path criterion. In panel c) we show that ammonia molecules do not network in the solutions we studied. An ammonia cluster is defined to be those ammonia molecules whose nearest neighbour fits the criteria N_A-$N_A \leq 3.8$ Å. It is observed that the 'clustering' is inside a percolation threshold [1] where $P = S^{-2.2}$) marked by the dashed green line.

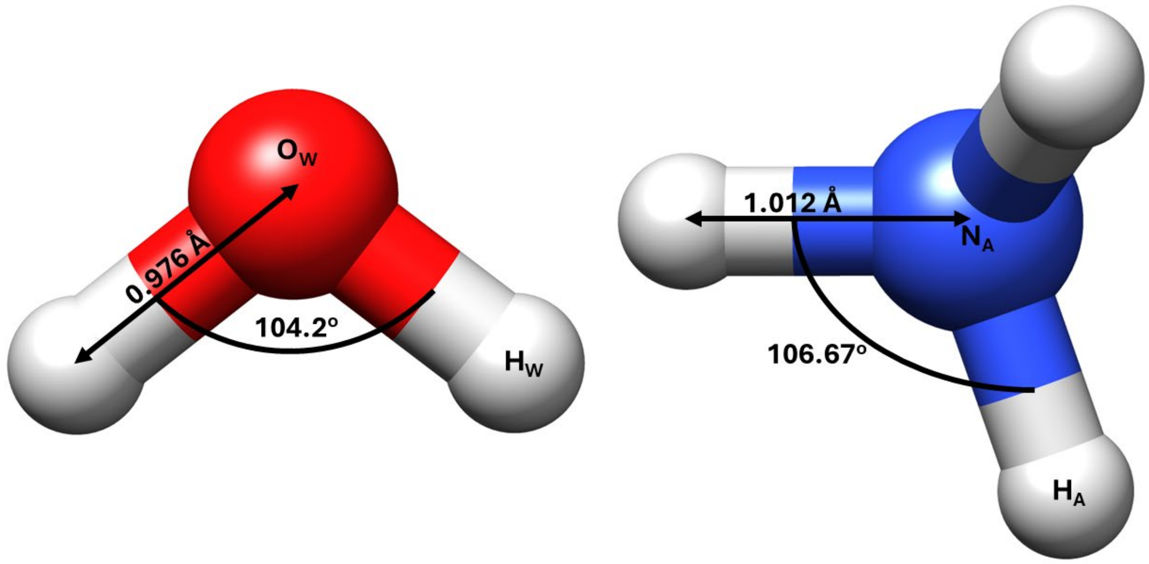

Fig. S3. The geometry of model water and ammonia molecules. The SPC/E model for water was adopted which features a bond angle of 109.47^0 and a bond length of 1.0 Å. This was changed to 104.2^0 and 0.976 Å in common with other diffraction studies [2]. For ammonia, the model of Gao et al.[3] was adopted.

Table S1. The intermolecular interactions of water molecules in aq. ammonia (273 K, 298 K), and pure water (298 K)

Conditions	Somogyi, 2005	Neish, 2009	Ramirez, 2010	Poch, 2012	Cleaves, 2014
Ammonia (wt.\%)	1,2	13	3.125, 6.25,12.5,25	25	15
Main text citation	48	4	49	50	51
Temp (K), 1 bar	298,373	253, 273, 297	96, 253, 277	253, 279	253

Table S2. The intermolecular interactions of water molecules in aq. ammonia (273 K, 298 K), and pure water (298 K)

Sample	Atom Pair	Å	Figure 3 Label	$CN_{(r(Å))}$
Water $_{298\ K}$	O_W-H_W	1.83	1	$0.51_{(1.80)}$
Am-Water $_{298\ K}$	O_W-H_W	1.80	1	$0.59_{(1.80)}$
Am-Water $_{273\ K}$	O_W-H_W	1.77	1	$0.67_{(1.80)}$
Water $_{298\ K}$	O_W-O_W	2.43	2	$1.94_{(2.40)}$
Am-Water $_{298\ K}$	O_W-O_W	2.37	2	$1.73_{(2.40)}$
Am-Water $_{273\ K}$	O_W-O_W	2.37	2	$1.81_{(2.40)}$
Water $_{298\ K}$	O_W-O_W	2.79	3	$1.21_{(2.76)}$
Am-Water $_{298\ K}$	O_W-O_W	2.73	3	$1.49_{(2.76)}$
Am-Water $_{273\ K}$	O_W-O_W	2.73	3	$1.72_{(2.76)}$
Water $_{298\ K}$	O_W-O_W	3.36	4	$4.42_{(3.30)}$
Am-Water $_{298\ K}$	O_W-O_W	3.30	4	$3.67_{(3.30)}$
Am-Water $_{273\ K}$	O_W-O_W	3.27	4	$3.68_{(3.30)}$
Water $_{298\ K}$	O_W-O_W	4.47	5	$12.00_{(4.50)}$
Am-Water $_{298\ K}$	O_W-O_W	4.50	5	$8.92_{(4.50)}$
Am-Water $_{273\ K}$	O_W-O_W	4.53	5	$9.04_{(4.50)}$

Table S3. The hydrogen bond energies of pure water (298 K), and aqueous ammonia a (273 K, 298 K).

Hydrogen bond	Atom pair	T(K)	Mean energy (kJ/Mol)
Pure Water	H_W-O_W	298	-18.01 ± 0.10
Bulk Water	H_W-O_W	298	-17.78 ± 0.24
Amm FSS water to water	H_W-O_W	298	-18.42 ± 0.25
Water to ammonia	H_W-N_A	298	-18.74 ± 0.26
Ammonia to water	H_A-O_W	298	-7.36 ± 0.12
Ammonia to ammonia	H_A-N_A	298	-6.94 ± 0.26
Bulk water	H_W-O_W	273	-18.18 ± 0.15
Amm FSS water to water	H_W-O_W	273	-18.60 ± 0.14
Water to ammonia	H_W-N_A	273	-18.89 ± 0.13
Ammonia to water	H_A-O_W	273	-7.48 ± 0.12
Ammonia to ammonia	H_A-N_A	273	-6.91 ± 0.19

Note: For cut-off distances see Table S3

Table S4. Lennard Jones parameters adopted for molecular models in EPSR. From Soper et al., Meersman et al, and Gao et al.[2–4] The experimental density of ammonia-water at 273 K was 0.102 atoms / Å^3 and at 298 K it was 0.100 atoms / Å^3.

Molecule	Formula	Atom type	No.	ε kJ mol^{-1}	σ Å	e e	Total e e
Water	H_2O	O_W	1	0.650	3.166	-0.8476	-0.8476
		H_W	2	0.000	0.000	0.4238	0.8476
Ammonia	NH_3	N_A	1	0.879	3.360	-1.026	-1.026
		H_A	3	0.000	0.000	0.342	1.026
							0.0000

Table S5: Cut-off distances for Hydrogen bond interactions in water and aqueous ammonia samples.

Atom Pair	Cut-off distance (Å)
N_A-N_A	3.6
N_A-O_W	3.6
O_W-O_W	3.3
H_A-O_W	2.5
O_W-H_W	2.4
N_A-H_W	2.4
N_A-H_A	2.4

Supporting Information Bibliography

1. Jan, N. Large lattice random site percolation. *Physica A: Statistical Mechanics and its Applications* **266**, 72–75 (1999).

2. Meersman, F., Bowron, D., Soper, A. K. & Koch, M. H. J. Counteraction of urea by trimethylamine N-oxide is due to direct interaction. *Biophys J* **97**, 2559–2566 (2009).

3. Gao, J., Xia, X. & George, T. F. Importance of bimolecular interactions in developing empirical potential functions for liquid ammonia. *Journal of Physical Chemistry* **97**, 9241–9247 (1993).

4. Soper, A. K. The radial distribution functions of water and ice from 220 to 673 K and at pressures up to 400 MPa. *Chem Phys* **258**, 121–137 (2000).
