# Supplementary material for: Solution structure of Titan-relevant aqueous ammonia by neutron diffraction
Source: Commun Chem. 2025 Aug 2;8:227. doi: 10.1038/s42004-025-01599-8 (PMC12318028; doi:10.1038/s42004-025-01599-8)
Supplement: Supplementary file 3 — Description of Additional Supplementary Files [file 42004_2025_1599_MOESM3_ESM.pdf]

Description of Additional Supplementary Files

File name- Supplementary Movie 1

File description- The spatial density distribution of nitrogen atoms of ammonia (blue) and oxygen atoms of water (red) around a central water molecule in ammonia-water (273 K). The surface contours contain the most probable (top 70%) locations for finding oxygen atoms and nitrogen atoms inside 3.5 Å. This data corresponds to a 360-degree rotation around the vertical y axis of the data presented in Fig.9 b.

File name- Supplementary Movie 2

File description- The spatial density distribution of nitrogen atoms of ammonia (blue) and oxygen atoms of water molecules (red) around a central ammonia molecule in ammonia-water (273 K). The surface contours contain the most probable (top 20%) locations for finding oxygen atoms or nitrgen atoms inside 4.0 Å. This data corresponds to a 360-degree rotation around the vertical y axis of the data presented in Fig.10 b.
